# Associations between prevalent multimorbidity combinations and prospective disability and self-rated health among older adults in Europe

**DOI:** 10.1186/s12877-019-1214-z

**Published:** 2019-07-27

**Authors:** Paige E. Sheridan, Christine A. Mair, Ana R. Quiñones

**Affiliations:** 1 Department of Family Medicine and Public Health, University of California, San Diego School of Medicine, San Diego, California USA; 2 Department of Public Health, San Diego State University School of Public Health, San Diego, California USA; 3 Department of Family Medicine and OHSU-PSU School of Public Health, Oregon Health & Science University, Portland, Oregon USA; 4 Department of Sociology & Anthropology, University of Maryland, Baltimore County, Baltimore, MD USA

**Keywords:** Multimorbidity, Multiple chronic conditions, Disability, Self-rated health, Depression

## Abstract

**Background:**

Multimorbidity is associated with greater likelihood of disability, health-related quality of life, and mortality, greater than the risk attributable to individual diseases. The objective of this study is to examine the association between unique multimorbidity combinations and prospective disability and poor self-rated health (SRH) in older adults in Europe.

**Methods:**

We conducted a prospective analysis using data from the Survey of Health, Ageing and Retirement in Europe in 2013 and 2015. We used hierarchical models to compare respondents with multiple chronic conditions to healthy respondents and respondents reporting only one chronic condition and made within-group comparisons to examine the marginal contribution of specific chronic condition combinations.

**Results:**

Less than 20% of the study population reported having zero chronic conditions, while 50% reported having at least two chronic conditions. We identified 380 unique disease combinations among people who reported having at least two chronic conditions. Over 35% of multimorbidity could be attributed to five specific multimorbidity combinations, and over 50% to ten specific combinations. Overall, multimorbidity combinations that included high depressive symptoms were associated with increased odds of reporting poor SRH, and increased rates of ADL-IADL disability.

**Conclusions:**

Multimorbidity groups that include high depressive symptoms may be more disabling than combinations that include only somatic conditions. These findings argue for a continued integration of both mental and somatic chronic conditions in the conceptualization of multimorbidity, with important implications for clinical practice and healthcare delivery.

**Electronic supplementary material:**

The online version of this article (10.1186/s12877-019-1214-z) contains supplementary material, which is available to authorized users.

## Background

Multimorbidity, most often defined as the presence of two or more co-existing chronic conditions, is highly prevalent among older adults across the world [1–5]. Multimorbidity is associated with greater likelihood of disability [6–10], major depressive disorder [11], lower self-rated health [12], quality of care [13] and increased healthcare utilization and cost [14, 15]. As the world’s population ages, there is a growing need for population-based research in multimorbidity to clarify the complex interactions between health-related quality of life, chronic disease and disability [16, 17].

Several seminal works have informed research and clinical practice to shift away from focusing on index, single, and individual chronic diseases and instead consider the full complement of chronic diseases as they co-occur in patients [1, 18, 19]. Yet, there is little consensus on how to measure and operationalize multimorbidity and quantify linkages between important geriatric syndromes and health-related quality of life consequences of multimorbidity among older adult populations. Specifically, gaps in this growing area of research involve increasing the evidence base on the epidemiology of multimorbidity and integrating patient-centered measures to assess the impact of multimorbidity on patients’ lives [20, 21].

An increasing number of studies have moved to identify specific multimorbidity patterns among older adults as an advancement over merely counting the total number of chronic conditions. A recent meta-analysis identified three nonrandom patterns of multimorbidity: musculoskeletal, cardiometabolic, and mental health [17]. Specific disease combinations may be more or less disabling than others, therefore it is clinically-relevant to assess outcomes for these combinations [22]. Further, depression is often co-morbid with other chronic conditions, but it is not standard practice to include depression in the operationalization of multimorbidity [23, 24]. While previous studies have examined the prevalence, correlates, and patterns of multimorbidity, and some studies examine the association between multimorbidity and depression [25–27], few have examined the effect of unique multimorbidity profiles, specifically those that incorporate depression into the operationalization of multimorbidity—which may have important and debilitating interactions with co-existing disease—and associations with health-related quality of life outcomes [7, 10, 28].

The purpose of this study is to assess the association between prevalent multimorbidity patterns, including chronic mental and somatic disease that occur in concert, on self-rated health and activities of daily living (ADL) and instrumental activities of daily living (IADL) disability using a cross-national sample of older adults in Europe. We compare respondents with multiple chronic conditions to healthy respondents and respondents reporting only one chronic condition and conduct within-group comparisons to examine the marginal conribution of specific chronic conditions. We discuss implications of unique multimorbidity patterns and examine country-by-country differences within multimorbidity groups. Examining specific multimorbidity patterns will allow for optimization of health care delivery and organization within clinical practice to improve care in these populations.

## Methods

The Survey of Health Ageing and Retirement in Europe (SHARE) is a multidisciplinary and cross-national longitudinal survey of noninstitutionalized Europeans aged 50 and over across 20 European countries and Israel. The target population consists of all persons aged 50 and over at the time of sampling and who have their permanent home in the respective SHARE country. Persons who are hospitalized, out of the country, or unable to speak the country’s language at the time of interview were excluded. Computer-assisted personal interviewing (CAPI) was used during face-to-face interviews. Recruitment strategies were country specific. If respondents faced physical or cognitive limitations, it is possible that the respondent is assisted by a proxy respondent. The full description of this survey has been published elsewhere [29].

The most recently available survey data from 2013 and 2015 (Waves 5 and 6) were used in this study. All covariates and exposure were assessed in 2013 and outcomes were assessed in 2015. 47,523 respondents were interviewed in both 2013 and 2015 sample waves, and we excluded 21,352 respondents who were under the age of 65 in 2013, and 862 respondents with one or more missing chronic disease responses. Seventeen respondents had non-positive survey weights and were excluded. Our final study population included 25,239 older adults. Fourteen countries were included in both the 2013 and 2015 SHARE survey: Austria, Germany, Sweden, Spain, Italy, France, Denmark, Switzerland, Belgium, Israel, Czech Republic, Luxembourg, Slovenia and Estonia.

### Disability assessment

SHARE respondents were asked about difficulty performing common everyday tasks to assess physical functioning, six activities of daily living, (ADL; dressing, walking across a room, bathing, eating, transferring from a bed, and toileting) and seven instrumental activities of daily living (IADL; using a map, meal preparation, grocery shopping, using a telephone, taking medication, doing work around the house, and managing money). The primary outcome variable is a combined ADL-IADL index variable assessed in 2015, created by summing the number of ADL and IADL disability counts for each respondent (range 0–13) [30].

### Self-rated health

Respondents were asked to rate their own health (SRH), on a scale from 0 to 4 (excellent, very good, good, fair, poor) [How would you rate your current health state?]. A higher score indicates poor SRH. The outcome variable is a dichotomous index variable assessed in 2015 for poor SRH (poor, fair) vs. good SRH (good, very good, and excellent).

### Chronic diseases

Respondents were asked about diagnoses of multiple chronic conditions: [Has a doctor ever told you that you had/Do you currently have any of the conditions on this card?]. We used the nine conditions queried in the survey that are associated with disability and poor SRH among older adults for this study: myocardial infarction, high blood pressure, stroke, diabetes, cancer, lung disease (excluding asthma), arthritis, Parkinson’s and Alzheimer’s diagnoses. Persons who indicated “yes” in Wave 5 or any previous wave were recorded as having the chronic condition, unless they disputed having the condition in a later wave. We additionally included a variable for self-reported high depressive symptomatology based on 12 questionnaire items. The instrument used was the EURO-D scale which includes 12 dichotomous indices: depression, pessimism, suicidality, guilt, sleep, interest, irritability, appetite, fatigue, concentration, enjoyment, and tearfulness [31]. Concordant with previous studies, respondents with four or more responses indicating depressive affect on the 12 question Euro-D scale (0–12) were defined as having high depressive symptomatology [31–33].

### Covariates

Sociodemographic covariates were assessed at baseline in 2013 and included age (years), gender (1 = female), education level (pre-primary, primary, lower secondary, upper secondary, post-secondary, 1st tertiary, 2nd tertiary), BMI calculated from interviewer measured height and weight, smoking (1 = current smoking), partnered (1 = married or cohabitating) and household net worth quartile in Euros adjusted for inflation.

## Statistical analyses

To assess the relationship between multimorbidity and disability (ADL-IADL index) and poor self-rated health, we examined the prevalence of combinations for participants who reported having at least two chronic conditions. The most prevalent multimorbidity combinations were tabulated and rank-ordered by percent of study participants in each multimorbidity combination. The ten most prevalent groups are examined to ensure sufficient sample size within each multimorbidity combination in regression analyses. Mean ADL-IADL index and mean SRH index were calculated for each multimorbidity group.

We estimated mixed negative binomial and mixed logistic regression models to assess the relationship between multimorbidity group and disability index and dichotomized poor self-rated health, respectively. ADL-IADL index and poor SRH reported in 2013 were included in respective models to account for baseline values. The associations in the present study should not be interpreted as causal associations, as we did not assess incident poor SRH and ADL-IADL index. Negative binomial models allow for modeling over-dispersed count data. Country was included as a random effect in all models to account for clustering of study participants by country of residence. We report incidence rate ratios (IRR) and odds ratios (OR) and 95% confidence intervals. A *p* value of < 0.01 was considered significant to account for multiple comparisons. Parameter estimates from negative binomial regression models are interpreted as the log of the ratio of expected counts, or the log of the rate ratio. These rate ratios can be interpreted as the relative difference in incidence rates between groups.

Across both dependent variables, we evaluated two different comparison groups: 1) healthy respondents who reported having no chronic conditions (*n* = 5,492) and 2) respondents who reported having only one chronic condition (*n* = 8,164). We further evaluated these associations among nested groups, or groups that included one additional condition to the existing combination to assess the relative impact of the additional condition between groups. For example: a group that includes both hypertension and arthritis and myocardial infarction would be compared with a group that includes only hypertension and arthritis to assess the relative contribution of myocardial infarction to the combination. An OR or IRR that is different from one in this context indicates that the additional condition contributes to a change in poor SRH or ADL-IADL index, while an OR or IRR that is close to one indicates no additional odds of reporting poor SRH or relative rate of ADL-IADL count associated with the addition of the given condition.

We present both unadjusted and adjusted analyses. Adjusted models include baseline self-rated health or ADL-IADL index, age, gender, education, BMI, current smoking, partnered status, and household net worth. All analyses were weighted using SHARE calibrated longitudinal survey weights to account for sampling. All denominators presented are unweighted and all percentages are weighted using these survey weights. Missing data for income and education was imputed using multiple imputations provided by SHARE [34]. All analyses were conducted using SAS 9.4.

## Results

Our study population consisted of 25,293 respondents across 14 countries and two survey waves in 2013 and 2015. 57% of participants were female and the mean age was 75. 11% were current smokers and 49% reported having two or more chronic conditions. The mean number of chronic conditions for the full sample was 1.68 and the mean ADL-IADL index was 0.81. 19% of the sample reported having zero chronic conditions, while 50% reported having at least two chronic conditions. 70% of the population reported no ADL-IADL limitations and 55% reported having “good” or better self-rated health. Hypertension was the most prevalent individual chronic condition (49%) followed by arthritis (34%) and high depressive symptoms (31%). Parkinson’s and Alzheimer’s were the least frequently reported individual chronic conditions. Baseline characteristics of the full study sample are summarized in Table 1.

**Table 1:** Baseline Characteristics of the Study Population, SHARE 2013–2015 (*n* = 25,293)

Characteristic	*N* (%)^a^ / Mean (SE)
Female	14,082 (56.82)
Age, mean	74.67 (0.13)
Body Mass Index, mean	26.82 (0.16)
Current Smoking	2,975 (11.13)
Higher Education	6,349 (19.71)
Partnered	17,090 (59.54)
Chronic conditions	
Myocardial Infarction	3,953 (15.42)
Hypertension	12,296 (48.76)
Stroke	1,229 (4.66)
Diabetes	4,025 (16.61)
Cancer	1,587 (7.05)
Lung Disease	1,708 (7.80)
High depressive symptoms	6,883 (30.5)
Parkinson’s	282 (1.11)
Arthritis	7,365 (34.08)
Alzheimer’s	395 (1.61)
Number of chronic diseases, mean	1.68 (0.04)
ADL & IADL index, mean	0.81 (0.06)
Self-Rated Health	
Excellent	1,499 (4.81)
Very Good	3,683 (11.31)
Good	9,169 (38.29)
Fair	8,067 (33.09)
Poor	2,875 (12.51)
Self-Rated Health, mean	2.37 (0.05)
Countries	
Austria	1,694 (2.34)
Germany	1,967 (26.89)
Sweden	2,258 (3.25)
Spain	2,863 (14.14)
Italy	2,049 (21.65)
France	1,726 (18.25)
Denmark	1,542 (1.77)
Switzerland	1,419 (2.74)
Belgium	2,113 (3.42)
Israel	899 (1.20)
Czech Republic	2,431 (3.18)
Luxembourg	465 (0.11)
Slovenia	1,232 (0.64)
Estonia	2,635 (0.42)

^a^Values are unweighted counts and weighted percentages

*ADL* Activities of daily living

*IADL* Instrumental activities of daily living

We identified 380 unique disease combinations in people who reported having at least two chronic conditions. Baseline study characteristics among participants with multimorbidity are shown in Additional file 1. Over 35% of multimorbidity could be attributed to five specific multimorbidity combinations, and over 50% to ten specific combinations. The top ten multimorbidity categories by prevalence among people who reported having at least two chronic conditions and their respective mean ADL-IADL counts and percent reporting poor SRH are reported in Table 2. Hypertension was present in eight of the ten groups, while high depressive symptomatology and arthritis were each included in six and five groups, respectively. Among individuals with at least two chronic conditions, 50% reported high depressive symptomatology.

**Table 2:** Mean SRH and ADL-IADL Index and 95% Confidence Interval of Study Population in 2015 by Multimorbidity Group, SHARE 2013–2015

		*N*^a^	%^b^	ADL-IADL (0–13)	Poor SRH
				Mean (95% CI)	% (SE)
Group 1	Hypertension + Arthritis	1274	21.4	0.71 (0.55, 0.87)	50.7 (3.0)
Group 2	Hypertension + High Depressive Symptoms	870	13.9	1.22 (0.90, 1.53)	54.8 (2.3)
Group 3	Arthritis + High Depressive Symptoms	649	13.6	1.56 (1.31, 1.80)	65.2 (3.1)
Group 4	Hypertension + Arthritis + High Depressive Symptoms	683	13.1	1.84 (1.54, 2.14)	72.4 (1.8)
Group 5	Hypertension + Diabetes Mellitus	801	12.3	0.97 (0.35, 1.58)	50.9 (4.1)
Group 6	Myocardial Infarction + Hypertension	572	7.3	0.81 (0.37, 1.25)	53.3 (3.1)
Group 7	Hypertension + Diabetes Mellitus + Arthritis + High Depressive Symptoms	250	5.6	2.05 (1.55, 2.55)	82.2 (5.1)
Group 8	Hypertension + Diabetes Mellitus + Arthritis	271	5.1	0.80 (0.63, 0.97)	69.8 (4.3)
Group 9	Hypertension + Diabetes Mellitus + High Depressive Symptoms	255	4.2	1.42 (0.59, 2.26)	72.2 (7.0)
Group 10	Myocardial Infarction + Hypertension + High Depressive Symptoms	250	3.4	1.45 (0.89, 2.01)	72.6 (4.2)

^a^Values are unweighted counts

^b^Values are weighted percentages

*ADL* Activities of daily living

*IADL* Instrumental activities of daily living

*SRH* Self rated health

**Fig. 1:**
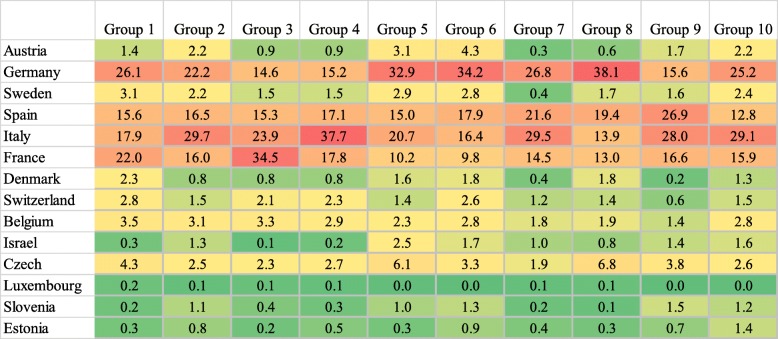
Multimorbidity Group Composition by Country, SHARE 2013–2015 (*n* = 11,644), Reported as Weighted Percentages

Figure 1 shows the top ten multimorbidity combinations by country of residence for the sample reporting at least two chronic conditions. Older adults in Italy represented the majority of individuals in five of the ten multimorbidity groups, and older adults in Germany represented the majority if individuals in four of the ten groups. France represented the majority in one multimorbidity group. Overall, Luxembourg, Slovenia, and Estonia had minority representation across all multimorbidity groups.

The results from unadjusted and adjusted negative binomial and logistic models are shown in Table 3. All ten multimorbidity groups were associated with significantly greater rates of ADL-IADL disability compared to healthy respondents with no chronic conditions in unadjusted models. In adjusted models, all groups except for Group 6 were associated with greater rates of ADL-IADL disability. When compared with respondents reporting only one condition, Groups 2–5, 7, 8 and 9 all had significantly greater ADL-IADL limitations, after adjustment.

**Table 3:** Logistic Regression and Negative Binomial Models of Poor Self-Rated Health and ADL-IADL Index on Multimorbidity Group, SHARE 2013–2015

	Reference Group: No Chronic Conditions (*n* = 5492)		Reference Group: One Chronic Condition (*n* = 8164)	
Poor SRH: OR (95% CI)	Unadjusted	Adjusted^a^	Unadjusted	Adjusted^a^
1: Hypertension + Arthritis	2.89 (2.55–3.28) *	1.58 (1.35–1.85) *	1.49 (1.32–1.67) *	1.19 (1.03–1.37)
2: Hypertension + High Depressive Symptoms	4.65 (4.01–5.40) *	1.99 (1.65–2.39) *	2.39 (2.07–2.76) *	1.50 (1.27–1.78) *
3: Arthritis + High Depressive Symptoms	4.73 (3.99–5.59) *	1.79 (1.45–2.22) *	2.43 (2.06–2.86) *	1.36 (1.12–1.66) *
4: Hypertension + Arthritis + High Depressive Symptoms	7.86 (6.59–9.37) *	2.17 (1.73–2.71) *	4.04 (3.4–4.79) *	1.71 (1.39–2.09) *
5: Hypertension + Diabetes Mellitus	2.84 (2.44–3.31) *	1.67 (1.39–2.01) *	1.46 (1.26–1.69) *	1.26 (1.06–1.49) *
6: Myocardial Infarction + Hypertension	4.67 (3.91–5.57) *	2.24 (1.80–2.79) *	2.40 (2.02–2.85) *	1.69 (1.37–2.07) *
7: Hypertension + Diabetes Mellitus + Arthritis + High Depressive Symptoms	15.66 (11.21–21.88) *	2.98 (2.01–4.41) *	8.05 (5.78–11.21) *	2.47 (1.70–3.59) *
8: Hypertension + Diabetes Mellitus + Arthritis	5.02 (3.90–6.45) *	1.65 (1.21–2.25) *	2.58 (2.01–3.3) *	1.29 (0.96–1.73)
9: Hypertension + Diabetes Mellitus + High Depressive Symptoms	7.96 (6.03–10.51) *	2.05 (1.46–2.87) *	4.09 (3.11–5.38) *	1.67 (1.22–2.31) *
10: Myocardial Infarction + Hypertension + High Depressive Symptoms	10.53 (7.82–14.2) *	2.43 (1.72–3.45) *	5.41 (4.03–7.27) *	1.95 (1.40–2.72) *
ADL/IADL: IRR (95% CI)	Unadjusted	Adjusted^b^	Unadjusted	Adjusted^b^
1: Hypertension + Arthritis	2.02 (1.66–2.47) *	1.50 (1.26–1.79) *	1.12 (0.95–1.32)	1.11 (0.96–1.29)
2: Hypertension + High Depressive Symptoms	3.48 (2.77–4.37) *	1.81 (1.48–2.21) *	1.93 (1.59–2.33) *	1.34 (1.13–1.58) *
3: Arthritis + High Depressive Symptoms	4.23 (3.27–5.46) *	2.00 (1.60–2.50) *	2.34 (1.89–2.9) *	1.45 (1.20–1.74) *
4: Hypertension + Arthritis + High Depressive Symptoms	4.59 (3.60–5.84) *	1.92 (1.55–2.38) *	2.54 (2.07–3.12) *	1.43 (1.20–1.70) *
5: Hypertension + Diabetes Mellitus	1.92 (1.48–2.49) *	1.76 (1.41–2.21) *	1.06 (0.86–1.31)	1.31 (1.10–1.58) *
6: Myocardial Infarction + Hypertension	2.09 (1.54–2.83) *	1.10 (0.85–1.43)	1.16 (0.91–1.48)	0.86 (0.70–1.07)
7: Hypertension + Diabetes Mellitus + Arthritis + High Depressive Symptoms	6.53 (4.36–9.80) *	2.31 (1.64–3.26) *	3.62 (2.59–5.05) *	1.86 (1.40–2.46) *
8: Hypertension + Diabetes Mellitus + Arthritis	2.13 (1.39–3.28) *	1.58 (1.10–2.27)	1.18 (0.84–1.67)	1.27 (0.95–1.70)
9: Hypertension + Diabetes Mellitus + High Depressive Symptoms	5.24 (3.46–7.93) *	2.58 (1.83–3.62) *	2.90 (2.07–4.06) *	2.00 (1.51–2.64) *
10: Myocardial Infarction + Hypertension + High Depressive Symptoms	5.12 (3.38–7.76) *	2.17 (1.56–3.02) *	2.84 (2.02–3.98) *	1.69 (1.29–2.23) *

^a^Adjusted for baseline self-rated health, age, sex, partnered, smoking status, BMI, education, net worth

^b^Adjusted for baseline ADL-IADL, age, sex, partnered, smoking status, BMI, education, net worth

* indicates *p* < 0 .01

*ADL* Activities of daily living

*IADL* Instrumental activities of daily living

*SRH* Self rated health

All ten multimorbidity groups had increased odds of reporting poor SRH when compared with healthy respondents and respondents with only one chronic condition in unadjusted models. In adjusted models, all groups were associated increased odds of reporting poor SRH when compared with healthy respondents. All groups were associated with increased odds of reporting poor SRH when compared with respondents with only one chronic condition, except for Group 8.

The head-to-head results from nested group comparisons are shown in Table 4. Unadjusted results are shown in Additional file 2. In nested comparisons, we examine perfect subsets of larger groups to evaluate the relative influence of one additional condition added to a multimorbidity combination. Nested comparisons are grouped by the condition that is added, assessing the marginal impact of the added condition. Groups 4, 7, 9 and 10 included high depressive symptoms added to the combinations represented in groups 1, 8, 5 and 6, respectively. After adjustment, all four of these groups had significantly greater ADL-IADL limitations compared with their relative comparison group that did not include high depressive symptoms. Groups 4, 7 and 9 had increased odds of reporting poor SRH when compared with their reference group that did not include high depressive symptoms. The addition of high depressive symptoms to hypertension and myocardial infarction (Group 10 compared to Group 6) did not result in significantly different odds of reporting poor SRH.

**Table 4:** Nested Comparisons: Adjusted Logistic Regression and Negative Binomial models of Poor Self-Rated Health and ADL-IADL Index on Multimorbidity Group, SHARE 2013–2015

Comparison Group	Reference Group	Poor SRH: OR (95% CI)^a^	ADL-IADL Index: IRR (95% CI)^b^
Addition of **High Depressive Symptoms** to Combination			
4: Hypertension + Arthritis + **High Depressive Symptoms**	1: Hypertension + Arthritis	1.44 (1.14–1.83) *	1.33 (1.12–1.59) *
9: Hypertension + Diabetes Mellitus + **High Depressive Symptoms**	5: Hypertension + Diabetes Mellitus	1.58 (1.11–2.24) *	1.74 (1.28–2.36) *
10: Myocardial Infarction + Hypertension + **High Depressive Symptoms**	6: Myocardial Infarction + Hypertension	1.24 (0.84–1.84)	1.90 (1.46–2.46) *
7: Hypertension + Diabetes Mellitus + Arthritis + **High Depressive Symptoms**	8: Hypertension + Diabetes Mellitus + Arthritis	2.15 (1.33–3.48) *	1.76 (1.31–2.38) *
Addition of **Diabetes Mellitus** to Combination			
8: Hypertension + **Diabetes Mellitus** + Arthritis	1: Hypertension + Arthritis	1.12 (0.82–1.54)	1.09 (0.83–1.44)
9: Hypertension + **Diabetes Mellitus** + High Depressive Symptoms	2: Hypertension + High Depressive Symptoms	1.13 (0.80–1.61)	1.42 (1.10–1.83) *
7: Hypertension + **Diabetes Mellitus** + Arthritis + High Depressive Symptoms	4: Hypertension + Arthritis + High Depressive Symptoms	1.44 (0.94–2.20)	1.30 (1.05–1.61)
Addition of **Arthritis** to Combination			
4: Hypertension + **Arthritis** + High Depressive Symptoms	2: Hypertension + High Depressive Symptoms	1.14 (0.88–1.46)	1.12 (0.93–1.33)
8: Hypertension + Diabetes Mellitus + **Arthritis**	5: Hypertension + Diabetes Mellitus	1.13 (0.82–1.56)	0.99 (0.71–1.38)
7: Hypertension + Diabetes Mellitus + **Arthritis** + High Depressive Symptoms	9: Hypertension + Diabetes Mellitus + High Depressive Symptoms	1.42 (0.87–2.32)	1.00 (0.77–1.29)
Addition of **Cardiovascular Conditions** to Combination			
10: **Myocardial Infarction** + Hypertension + High Depressive Symptoms	2: Hypertension + High Depressive Symptoms	1.39 (0.96–2.01)	1.35 (1.06–1.72)
4: **Hypertension** + Arthritis + High Depressive Symptoms	3: Arthritis + High Depressive Symptoms	1.28 (0.98–1.68)	1.00 (0.84–1.20)

^a^ Adjusted for baseline self-rated health, age, sex, partnered, smoking status, BMI, education, net worth

^b^ Adjusted for baseline ADL-IADL, age, sex, partnered, smoking status, BMI, education, net worth

* indicates *p* < 0 .01

*ADL* Activities of daily living

*IAD*L Instrumental activities of daily living

*SRH* Self rated health

The bold term is the condition that is added to the combination relative to the reference group

All other nested comparisons included the addition of a somatic condition in their comparison group. None of these groups had significantly different odds of reporting poor SRH. All but one of the groups had no change in rates of ADL-IADL disability. The addition of diabetes mellitus to hypertension and high depressive symptoms (Group 9 compared to Group 2) was associated with significantly greater ADL-IADL disability.

## Discussion

This study examined associations between the ten most prevalent multimorbidity groups with disability and poor self-rated health. Multimorbidity was highly prevalent in this sample of older Europeans, with 50% of the sample reporting at least two chronic conditions. About 48% of the sample reported poor or fair SRH, and 30% reported at least one ADL/IADL limitation. Overall, high depressive symptomatology was identified in 50% of older adults with multimorbidity. Relative to older adults in Europe with one or no chronic conditions, older adults in all multimorbidity groups reported increased disability burden and increased odds of poor self-rated health. Hypertension was present in nearly all of the most prevalent combinations, and high depressive symptomatology and arthritis were present in a majority of the most prevalent combinations. This population had similar rates of multimorbidity when compared with studies involving US and Australian older adults [6, 35], and in other studies of older European adults [15].

We find that multimorbidity group combinations are not distributed evenly across nations, likely reflecting differences in both the demographic composition and multimorbidity burdens across the included European countries. The clustering of multimorbidity categories in certain counties, such as Italy and Germany, is indicative of underlying population and demographic compositional characteristics, as well as the differential distribution of risk factors and chronic disease etiology among European countries and region-specific differences in ascertainment and diagnoses of chronic conditions. Further exploration of specific multimorbidity patterns within European countries is warranted.

Multimorbidity combinations that include high depressive symptoms are associated with high rates of prospective ADL-IADL disability, and increased odds of reporting poor SRH in this study. It is plausible that, on average, the addition of high depressive symptoms to any chronic condition or combination of chronic conditions may be more disabling or associated with worse SRH than the addition of an individual somatic condition. When we examine nested multimorbidity groups, we find that all four groups that include high depressive symptoms have higher ADL-IADL burden, and three of the four groups have increased odds of reporting poor SRH. Concurrently, when we examine nested groups that only include the addition of a somatic condition, we found no differences in odds of reporting poor SRH. The ORs in these comparisons are close to one, possibly indicating that the addition of a somatic condition alone to an individual with high depressive symptoms has a relatively small effect on SRH. We see a similar pattern when looking at ADL-IADL index, where almost all groups that include the addition of a somatic condition do not have significantly different rates of ADL-IADL disability, with IRR close to one. However, we do find increased rates of ADL-IADL disability in one somatic condition group, the comparison of Group 9 with Group 2, where diabetes mellitus is added to high depressive symptoms and hypertension.

Taken together, these results suggest that the addition of high depressive symptomatology alone may be considerably more disabling than the addition of another somatic condition. These findings are consistent with previous studies in the US that conceptualized multimorbidity combining somatic and mental conditions [7, 22]. The present study provides potential evidence that this association extends cross-nationally. To our knowledge this is one of the first studies to examine associations between unique multimorbidity combinations and associations with prospective self-rated health and ADL-IADL disability in Europe. Our analysis of a large, cross-nationally representative, longitudinal dataset allows us to assess associations while accounting for prior SRH and ADL-IADL disability to account for temporal sequence. Most importantly, these findings underscore the potential multiplicative effect chronic conditions may have on SRH and disability and highlight the potential role of high depressive symptomatology as a key co-morbid condition in the operationalization of multimorbidity.

This study has several noteworthy limitations. First, the use of self-reported measures of chronic health conditions and health states may under or over-estimate the true prevalence of these conditions in this older population, particularly among lower socioeconomic and lower education groups [36]. However, several studies have shown reasonable concordance between participant reports of physician-diagnosed conditions and administrative and medical record data sources [37, 38]. Second, condition severity could not be assessed and may be an important consideration in comparing multimorbidity combinations and associations with the outcomes of interest. However, chronic disease severity is notoriously difficult to capture with high reliability in population-based data sources [39] . In sensitivity analyses, we observed that less than 1% of participants who reported having a chronic condition in wave 5 reported no longer having that condition in wave 6, which would indicate a possible misunderstanding about the presence or absence of the condition. Still, it is important to assess self-report of chronic conditions, because they represent individuals’ beliefs about the chronic diseases they have and dictate self-management behaviors. Future research should further clarify chronic disease status and ascertainment in longitudinal studies—possibly with the inclusion of information treatments and medication use to confirm diagnoses present—to better assess the development and progression of multimorbidity over time [40]. In addition, better specifying the psychosocial risk factors (and protective, buffering factors) associated with multimorbidity is an important area of research that should be prioritized in future studies.

## Conclusions

This is the first cross-national study to examine the association between unique multimorbidity combinations and ADL-IADL disability and self-rated health. We find that multimorbidity is highly prevalent among European older adults and is associated with higher rates of disability and increased odds of poor self-rated health. Finally, multimorbidity combinations that include high depressive symptoms may be more disabling than combinations that include only somatic conditions. These findings argue for a continued integration of both mental as well as somatic chronic conditions in the conceptualization of multimorbidity, with important implications for clinical practice and healthcare delivery. Organizing health care delivery to better address the multiplicative effect of the presence of multiple chronic conditions, particularly depression, should be prioritized. European health systems may want to emphasize management of chronic mental health conditions for patients with multiple chronic conditions to improve health outcomes associated with aging.

## Additional files

Additional file 1: Baseline Characteristics of the Study Population with Two or More Chronic Conditions, SHARE 2013–2015 (*n* = 11,644). (DOCX 17 kb)

Additional file 2: **Table S4.** Nested Comparisons: Unadjusted Logistic Regression and Negative Binomial models of Poor Self-Rated Health and ADL-IADL Index on Multimorbidity Group, SHARE 2013–2015. (DOCX 17 kb)

## Data Availability

The datasets generated and/or analyzed during the current study are available in the Survey of Health Ageing and Retirement repository, http://www.share-project.org, [Börsch-Supan, A. (2018). *Survey of Health, Ageing and Retirement in Europe (SHARE) Wave 5*. Release version: 6.1.0. SHARE-ERIC. Data set. DOI: 10.6103/SHARE.w5.611; Börsch-Supan, A. (2018). *Survey of Health, Ageing and Retirement in Europe (SHARE) Wave 6*. Release version: 6.1.0. SHARE-ERIC. Data set. DOI: 10.6103/SHARE.w6.611].

## Abbreviations

ADL: Activities of daily living
CAPI: Computer-assisted personal interviewing
IADL: Instrumental activities of daily living
SRH: Self rated health
