# Supplementary material for: Associations between prevalent multimorbidity combinations and prospective disability and self-rated health among older adults in Europe
Source: BMC Geriatr. 2019 Jul 27;19:198. doi: 10.1186/s12877-019-1214-z (PMC6661084; doi:10.1186/s12877-019-1214-z)
Supplement: Supplementary file 1 — Baseline Characteristics of the Study Population with Two or More Chronic Conditions, SHARE 2013–2015 (n = 11,644). (DOCX 17 kb) [file 12877_2019_1214_MOESM1_ESM.docx]

Additional file 1

| **Baseline Characteristics of the Study Population with Two or More Chronic Conditions, SHARE 2013 - 2015 (n=11,644)** | |
| --- | --- |
| Characteristic | N (%)^a^ / Mean (SE) |
| Female | 7110 (62.75) |
| Age, mean | 75.88 (0.15) |
| Body Mass Index, mean | 27.59 (0.18) |
| Current Smoking | 1193 (9.17) |
| Higher Education | 2413 (15.14) |
| Partnered | 7361 (54.57) |
| Chronic conditions |  |
| Myocardial Infarction | 3393 (26.93) |
| Hypertension | 8590 (72.23) |
| Stroke | 1120 (8.64) |
| Diabetes | 3449 (28.84) |
| Cancer | 1292 (11.92) |
| Lung Disease | 1456 (13.61) |
| High depressive symptoms | 5800 (53.1) |
| Parkinson’s | 243 (2) |
| Arthritis | 5860 (55.42) |
| Alzheimer’s | 358 (2.87) |
| Number of chronic diseases, mean | 2.33 (0.02) |
| ADL & IADL index, mean | 1.63 (0.09) |
| Self-Rated Health |  |
| Excellent | 170 (1.34) |
| Very Good | 742 (4.91) |
| Good | 3258 (28.03) |
| Fair | 4958 (44.84) |
| Poor | 2516 (20.88) |
| Self-Rated Health, mean | 2.79 (0.03) |
| Countries |  |
| Austria | 664 (1.89) |
| Germany | 965 (27.88) |
| Sweden | 863 (2.6) |
| Spain | 1418 (15.07) |
| Italy | 993 (22.16) |
| France | 870 (18.78) |
| Denmark | 611 (1.44) |
| Switzerland | 417 (1.67) |
| Belgium | 890 (2.86) |
| Israel | 451 (1.07) |
| Czech Republic | 1312 (3.41) |
| Luxembourg | 252 (0.12) |
| Slovenia | 527 (0.56) |
| Estonia | 1411 (0.46) |

^a^Values are unweighted counts and weighted percentages
