# Supplementary material for: Associations between prevalent multimorbidity combinations and prospective disability and self-rated health among older adults in Europe
Source: BMC Geriatr. 2019 Jul 27;19:198. doi: 10.1186/s12877-019-1214-z (PMC6661084; doi:10.1186/s12877-019-1214-z)
Supplement: Supplementary file 2 — Table S4. Nested Comparisons: Unadjusted Logistic Regression and Negative Binomial models of Poor Self-Rated Health and ADL-IADL Index on Multimorbidity Group, SHARE 2013–2015. (DOCX 17 kb) [file 12877_2019_1214_MOESM2_ESM.docx]

Additional file 2

| **Table 4. Nested Comparisons: Unadjusted Logistic Regression and Negative Binomial models of Poor Self-Rated Health and ADL-IADL Index on Multimorbidity Group, SHARE 2013-2015** | | | |
| --- | --- | --- | --- |
| Comparison Group | Reference Group | Poor SRH:  OR (95% CI) | ADL-IADL Index:  IRR (95% CI) |
| Addition of **High Depressive Symptoms** to Combination | | | |
| 4: Hypertension + Arthritis + **High Depressive Symptoms** | 1: Hypertension + Arthritis | 2.72 (2.23 - 3.31) * | 2.27 (1.87 - 2.75) * |
| 9: Hypertension + Diabetes Mellitus + **High Depressive Symptoms** | 5: Hypertension + Diabetes Mellitus | 2.80 (2.07 - 3.79) * | 2.73 (1.99 - 3.75) * |
| 10: Myocardial Infarction + Hypertension + **High Depressive Symptoms** | 6: Myocardial Infarction + Hypertension | 2.26 (1.61 - 3.16) * | 2.45 (1.80 - 3.33) * |
| 7: Hypertension + Diabetes Mellitus + Arthritis + **High Depressive Symptoms** | 8: Hypertension + Diabetes Mellitus + Arthritis | 3.12 (2.07 - 4.70) * | 3.06 (2.28 - 4.12) * |
| Addition of **Diabetes Mellitus** to Combination | | | |
| 8: Hypertension + **Diabetes Mellitus** + Arthritis | 1: Hypertension + Arthritis | 1.73 (1.33 - 2.27) * | 1.06 (0.78 - 1.42) |
| 9: Hypertension + **Diabetes Mellitus** + High Depressive Symptoms | 2: Hypertension + High Depressive Symptoms | 1.71 (1.26 - 2.31) * | 1.51 (1.13 - 2.00) * |
| 7: Hypertension + **Diabetes Mellitus** + Arthritis + High Depressive Symptoms | 4: Hypertension + Arthritis + High Depressive Symptoms | 1.99 (1.38 - 2.88) * | 1.42 (1.12 - 1.82) * |
| Addition of **Arthritis** to Combination | | | |
| 4: Hypertension + **Arthritis** + High Depressive Symptoms | 2: Hypertension + High Depressive Symptoms | 1.69 (1.36 - 2.09) * | 1.32 (1.08 - 1.61) * |
| 8: Hypertension + Diabetes Mellitus + **Arthritis** | 5: Hypertension + Diabetes Mellitus | 1.76 (1.33 - 2.34) * | 1.11 (0.79 - 1.56) |
| 7: Hypertension + Diabetes Mellitus + **Arthritis** + High Depressive Symptoms | 9: Hypertension + Diabetes Mellitus + High Depressive Symptoms | 1.97 (1.29 - 3.01) * | 1.25 (0.94 - 1.66) * |
| Addition of **Cardiovascular Conditions** to Combination | | | |
| 10: **Myocardial Infarction** + Hypertension + High Depressive Symptoms | 2: Hypertension + High Depressive Symptoms | 2.26 (1.64 - 3.12) * | 1.47 (1.11 - 1.95) * |
| 4: **Hypertension** + Arthritis + High Depressive Symptoms | 3: Arthritis + High Depressive Symptoms | 1.66 (1.32 - 2.09) * | 1.08 (0.88 - 1.33) |

*indicates p <0.01
